# Developing adaptive interventions for adolescent substance use treatment settings: protocol of an observational, mixed-methods project

**DOI:** 10.1186/s13722-017-0099-4

**Published:** 2017-12-19

**Authors:** Sean Grant, Denis Agniel, Daniel Almirall, Q. Burkhart, Sarah B. Hunter, Daniel F. McCaffrey, Eric R. Pedersen, Rajeev Ramchand, Beth Ann Griffin

**Affiliations:** 1 RAND Corporation, 1776 Main Street, Santa Monica, CA 90407 USA; 2 Institute for Social Research, University of Michigan, 426 Thompson Street, Ann Arbor, MI 48104-2321 USA; 3 Educational Testing Service, 660 Rosedale Road, Princeton, NJ 08541 USA; 4 RAND Corporation, 1200 South Hayes Street, Arlington, VA 22202-5050 USA

**Keywords:** Adaptive interventions, Substance use treatment, Clinical decision-making, Adolescents, Alcohol, Drugs

## Abstract

**Background:**

Over 1.6 million adolescents in the United States meet criteria for substance use disorders (SUDs). While there are promising treatments for SUDs, adolescents respond to these treatments differentially in part based on the setting in which treatments are delivered. One way to address such individualized response to treatment is through the development of adaptive interventions (AIs): sequences of decision rules for altering treatment based on an individual’s needs. This protocol describes a project with the overarching goal of beginning the development of AIs that provide recommendations for altering the setting of an adolescent’s substance use treatment. This project has three discrete aims: (1) explore the views of various stakeholders (parents, providers, policymakers, and researchers) on deciding the setting of substance use treatment for an adolescent based on individualized need, (2) generate hypotheses concerning candidate AIs, and (3) compare the relative effectiveness among candidate AIs and non-adaptive interventions commonly used in everyday practice.

**Methods:**

This project uses a mixed-methods approach. First, we will conduct an iterative stakeholder engagement process, using RAND’s ExpertLens online system, to assess the importance of considering specific individual needs and clinical outcomes when deciding the setting for an adolescent’s substance use treatment. Second, we will use results from the stakeholder engagement process to analyze an observational longitudinal data set of 15,656 adolescents in substance use treatment, supported by the Substance Abuse and Mental Health Services Administration, using the Global Appraisal of Individual Needs questionnaire. We will utilize methods based on Q-learning regression to generate hypotheses about candidate AIs. Third, we will use robust statistical methods that aim to appropriately handle casemix adjustment on a large number of covariates (marginal structural modeling and inverse probability of treatment weights) to compare the relative effectiveness among candidate AIs and non-adaptive decision rules that are commonly used in everyday practice.

**Discussion:**

This project begins filling a major gap in clinical and research efforts for adolescents in substance use treatment. Findings could be used to inform the further development and revision of influential multi-dimensional assessment and treatment planning tools, or lay the foundation for subsequent experiments to further develop or test AIs for treatment planning.

**Electronic supplementary material:**

The online version of this article (10.1186/s13722-017-0099-4) contains supplementary material, which is available to authorized users.

## Background

Over 6% of all 12–17 year olds (or 1.6 million youth) meet criteria for a substance use disorder (SUD) [1]. SUDs can lead to immediate and long-term effects for adolescents, such as poor school performance, school drop-out and delinquency, future health problems, motor vehicle accidents, unintentional injuries, and suicide [2–5]. Although many adolescents with SUDs receiving treatment improve in the short-term, the vast majority continue to use or experience substance-related consequences within 6–12 months of discharge [7–11], while a large percentage dropout from treatment prior to finishing a particular treatment episode [12, 13].

Adolescents respond to treatment differentially in part based on the treatment setting: that is, whether care is received in outpatient, intensive outpatient, inpatient, or residential settings [6, 14–17]. A sequential, individualized approach to making decisions about substance use treatment aims to address heterogeneity in responses to treatment across adolescents as well as for the same adolescent over time [15, 18, 19]. In this approach, treatment providers make decisions about (1) what treatment to provide initially, (2) how best to monitor response to treatment, and (3) whether or how best to alter treatment [20–22]. There is currently little empirical research on which settings to offer treatment, at what stages in the clinical pathway, based on what kind of treatment response, measured using which variables—and little guidance on such *sequential* decision-making as a result. Thus, addiction science and clinical practice would benefit from empirically-based guidance for altering treatment setting of adolescents with SUDs.

### Adaptive interventions for adolescent substance use treatment settings

*Adaptive interventions* (AIs)—also known as adaptive treatment strategies, treatment algorithms, or dynamic treatment regimens—assist sequential, individualized decision-making by recommending whether, how, or when to alter treatment for an individual at critical decision points [23–25]. While AIs can be developed for clinical treatments or adherence interventions [26, 27], this project focuses specifically on developing AIs for adolescent substance use treatment *settings*: i.e., well-operationalized, empirically-supported recommendations to consider when making individualized, sequential decisions about which treatment setting an adolescent with an SUD should receive treatment, based on their individual needs. These AIs will be composed of (1) critical decision stages (i.e., 3-month intervals), (2) setting options, (3) tailoring variables and outcomes used to make decisions concerning which service to provide, and (4) replicable decision rules linking treatment setting recommendations, responses to tailoring variables, and desired outcomes. These AIs can assist families, providers, and policymakers in deciding on treatment settings for adolescent clients at multiple stages over time.

**Fig. 1:**
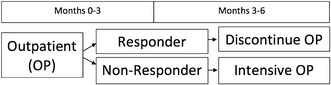
Example of a 6-month, two-stage service-level adaptive intervention (AI) for adolescent marijuana users

As an example, consider the AI shown in Fig. 1. There are two decision points: stage 1 concerns which service to recommend for months 0–3, and stage 2 concerns which service to recommend during months 3–6. In this example, there is only a single service option at stage 1, namely, outpatient services assigned at entry. In stage 2, there are two services options: discontinue outpatient or switch to intensive outpatient treatment. There are no tailoring variables at stage 1, while the tailoring variable at stage 2 is “responder status” during months 0–3 (e.g., abstinent or not during months 0–3). More complex AIs may include more than two stages, intervention options at each stage, and tailoring variables.

Although there is research related to AIs for SUD treatment [15, 28–31], we are unaware of any research on recommendations for the setting of adolescent substance use treatment. The American Society of Addiction Medicine (ASAM) Criteria [32] are a major step forward in comprehensive, individualized treatment planning, although the research foundations for the ASAM Criteria are based largely on adult data [33–40]. Additional research is needed to generate hypotheses about, and to better understand the utility of proposed AIs, for *adolescents.* This project aims to develop and evaluate AIs that begin to provide much-needed empirical evidence addressing important questions such as: What tailoring variables and outcomes should be considered when deciding an adolescent’s treatment setting? What values on these tailoring variables signify response versus non-response? Are there any combinations of initial and subsequent treatment setting that work synergistically or antagonistically?

### Methods for developing AIs using observational, practice-based data

There are several methods for making causal inferences about the effects of AIs that could be applied to observational, practice-based data [41, 42]. However, there have been few applications of such methods to complex, practice-based observational datasets relevant to adolescent SUDs. We suspect that methods available to identify AIs have not been readily adopted to observational data analyses in adolescent SUD in part due to a lack of theory and conceptual models to guide the development of AIs [54]. As such, the current study relies on a mixed methods approach using stakeholder engagement to compliment statistical methods used to inform the identification of potentially meaningful AIs. In addition, an empirical study of the causal effects of AIs using observational study data particularly requires careful consideration of the potential impact of time-varying confounding bias [43–50]. In the sequential decision-making setting, confounders of the effect of subsequent treatment are often also outcomes of previous treatment. In such settings, the use of standard regression, or even naïve use of standard propensity score adjustments, may actually cause more bias [51–53]. Appropriate and robust inverse-probability-of-treatment weighting methods can account for such confounders.

### Objectives

This study protocol describes a project to begin the development of AIs for the settings of adolescent substance use treatment, based on adolescents’ evolving individual needs throughout the clinical pathway. This project has three discrete aims. First, we aim to explore the views of various stakeholders (parents, providers, policymakers, and researchers) on important factors to consider when deciding the setting of substance use treatment for adolescents. Second, we aim to generate hypotheses concerning candidate AIs via statistical analyses of observational, practice-based empirical data from adolescent substance use treatment settings and informed by our examination of stakeholder views. Third, we aim to compare the relative effectiveness among candidate AIs as well as non-adaptive interventions that are commonly used in everyday practice. The empirically-informed AIs we aim to produce can help guide future placement of adolescents with SUDs into the most appropriate treatment settings at the most appropriate times for their individual needs, as well as lay the foundations for future randomized trials to more rigorously evaluate or identify effective AIs [23, 55–63].

## Methods

### Global Appraisal of Individual Needs

The current project will utilize longitudinal observational data on 15,696 unique adolescents who were administered the Global Appraisal of Individual Needs (GAIN) [64]. The GAIN has eight sections assessing background and demographic characteristics, substance use, physical health, mental health, risk behaviors, environmental, legal, and educational/vocational problem areas. Within each problem area, items assess problem characteristics, recency, severity, and service utilization. As such, use of longitudinal GAIN data provides the current study with an opportunity to identify and assess the relative effectiveness of candidate AIs. The data was routinely collected by Center for Substance Abuse Treatment (CSAT) discretionary grantees engaged in adolescent substance use program activities between 1997 and 2012. During this time, recipients of CSAT discretionary grants for adolescent substance use treatment research collected data on their clients using the GAIN. Adolescents were surveyed at four time points over the course of a year (namely, at intake and at 3-, 6-, and 12-months post-intake). Gender and race distributions closely mirrored those found in adolescent treatment samples nationally: namely, 74% of admissions were male, 18% were non-Hispanic African American, and 35% were Hispanic/Latino. It is important to note that this dataset is not necessarily representative of all adolescent substance use treatment facilities operating today—we suspect these are better performing facilities [65]. However, in the absence of such a large nationally-representative dataset, this one is uniquely poised to address our project aims.

### Aim 1. Identify key components of feasible AIs

#### Objectives

The first aim of this project is to examine the views of stakeholders (parents, clinicians, researchers, policymakers) on which tailoring variables and outcomes are most important to consider when deciding the setting of substance use treatment for adolescents. This aim will be used to inform the empirical data analyses for the remaining project aims.

#### Online Delphi process

Using an online system called ExpertLens [66], we will conduct an online Delphi process [67] in which we will ask stakeholders to rate the importance of individual needs contained within the GAIN as potential tailoring variables for treatment or key outcomes of interest. We will organize these individual needs according to the six dimensions of the ASAM Criteria: (1) acute intoxication and withdrawal potential; (2) biomedical conditions and complications; (3) emotional/behavioral/cognitive conditions and complications; (4) readiness to change; (5) relapse, continued use, or continued problem potential; and (6) recovery/living environment. We will also ask participants to rate the importance of clinical outcomes based on the National Outcome Measures (NOMs) from the Substance Abuse and Mental Health Services Administration (SAMHSA) [68].

Potential participants will be identified by first constructing a list based on published research, suggestions from known stakeholders in this area, and member lists of relevant societies and organizations. This will be followed by “snowball sampling” in which stakeholders can nominate further participants. To be eligible, participants must identify with one of the following four stakeholder groups: (1) parents of adolescents who have received substance use treatment; (2) providers of adolescent substance use treatment; (3) professionals involved in program planning at the clinic, health-system, state, or federal levels; or (4) researchers of adolescent substance use treatment. We aim to recruit at least 20–40 participants per stakeholder group. To improve recruitment and retention, participants will receive a $200 gift card for completing the process.

Our Delphi process will involve two separate rating rounds, with a round of online group discussion and feedback in between. We expect to keep each round open for 7–10 days and for each round to take participants about 1 h to complete. In Round One, participants will use a 9-point Likert scale to rate the importance of assessing each need or goal for deciding the appropriate setting (outpatient, intensive outpatient, residential, or inpatient) for an adolescent’s substance use treatment. A rating of 1 will correspond to “of lower importance” and a rating of 9 to “of higher importance.” Participants will also be able to comment on the rationale underpinning their ratings as well as suggest additional individual needs and treatment goals to rate in Round Three.

In Round Two, participants will see graphed results from Round One—including the median and frequencies for each item—as well as how a participant’s own rating compares to group ratings. They will also be shown decisions about the group’s agreement or disagreement on the importance of items for the checklist, as determined by the inter-percentile range adjusted for symmetry (IPRAS) analysis technique from the RAND/UCLA Appropriateness method [69]. If an item has disagreement, it is considered to have uncertain importance. If an item has agreement, the tertile in which the median rating for importance falls will be analyzed: a median score between 1 and 3 will indicate lower importance, 4 and 6 will indicate moderate importance, and 7 and 9 will indicate higher importance. These determinations will be summarized in user-friendly, color-coded text beneath each graph: green text indicating the group agreed the item has higher importance; yellow text, that the item has moderate importance; and red text, that the item has lower importance. Participant comments on each item from Round One will also be provided so participants can understand others’ rationales for their ratings. Participants will be asked to discuss these results in an anonymous online discussion forum to explore areas of agreement and disagreement. Rather than having participants sign in simultaneously, they post comments at their own leisure at times convenient to their schedules to increase engagement in discussion while preventing participant fatigue. Project team members will moderate all panels to promote participant engagement in discussions.

In Round Three, participants will re-rate each item in light of Round Two discussions of Round One results, as well as rate new items participants may have proposed in Round One. The process allows participants to revise their views and identify items they deem most important for the checklist. On the last page of the Round Three questionnaire, participants will be asked to provide input on the ExpertLens process. Participant anonymity of responses will be ensured via use of usernames in the ExpertLens system (e.g., Participant 1, Participant 2, etc.). Individual responses will be known only to the ExpertLens moderators.

Upon completion of the Delphi process, a descriptive analysis of participant ratings will characterize the distribution of group responses from each round, estimate changes in group responses between rounds, and determine areas of agreement and disagreement. Those items reaching consensus for high importance (using the IPRAS method) in Round Three will be prioritized for inclusion in the decision rules to be developed and evaluated in later project stages. To interpret these ratings, a thematic analysis will be conducted by systematically coding all comments linked to each of the items, indexing codes into preliminary and inductively identified themes, charting and integrating themes across items, and relabeling the final themes as appropriate [70].

### Aim 2. Empirically identify high-quality candidate AIs

#### Objective

The second aim of this project is to generate hypotheses about candidate AIs for deciding the setting for an adolescent’s substance use treatment using practice-based observational data on the GAIN from the CSAT dataset [64]. The development of candidate AIs will be informed by our stakeholder engagement and will consist of recommendations at two decision points: 0–3 months (stage 1 decision) and 3–6 months (stage 2 decision).

#### Settings of adolescent substance use treatment

The two stages of treatment (0–3 and 3–6 months) were determined by the GAIN observational study data collection protocol. For decisions about adolescent substance use treatment settings (i.e., residential vs. outpatient vs. intensive outpatient treatment), the 3-month time interval in the CSAT data is highly relevant, as participation in treatment services for at least 90 days is generally regarded as a best practice [6, 8, 71], and most evidence-based prevention/treatment programs for substance use disorders among adolescents are an average of 12 weeks long [10, 16, 72].

At each stage, there are four possible decisions regarding treatment setting: outpatient, intensive outpatient, residential/inpatient, or no treatment. Outpatient settings involve organized services for substance use that do not require an adolescent to be admitted to a residential program or hospital. Intensive outpatient settings require additional structure and support than regular outpatient services. Residential settings involve intensive, structured services for substance use that require an adolescent to be admitted but do not involve hospitalization. Inpatient settings involve intensive, structured services for substance use that require 24-h care in a safe and secure hospital unit. In our dataset, outpatient settings are the most common (see Table 1). In this study, inpatient and residential treatment settings are combined because they are asked about jointly in the GAIN survey.

**Table 1:** Number of youth in each treatment service

Time period	Level of care grouped		
	OP	IOP	RES/IP
Between intake and 3-month	8518	1800	1743
Between 3 and 6-months	4898	1137	1225

Adolescent participation in a treatment setting during each 90-day period (“stage”) will be assessed using a combination of treatment log data and adolescents’ self-reports of treatment experiences in the 90 days prior to a follow-up visit. Our treatment measures group adolescents into the four specific treatment settings based on whether or not an adolescent received any amount of treatment in the specific setting during the 90-day period in question. For example, to be in the residential group, an adolescent only needs to have received treatment in a residential setting for at least 1 day during the past 90 days. Adolescents in the “no treatment” condition will be coded as such if they reported no days of treatment received in outpatient, intensive outpatient, or residential/inpatient settings during the past 90 day period. We will also conduct sensitivity analyses assigning adolescents to the treatment setting in which they spent the most days over the 90 day period.

#### Candidate tailoring and confounding variables

Overall, the GAIN contains over 1000 items and 100-symptom count, change score, and service utilization indices that could be used for defining AIs and for dealing with confounding adjustment over time [73]. Moreover, since the GAIN was developed to assist clinicians with patient placement decisions, over 80 items were designed to operationalize the ASAM Criteria [74, 75]. Given the structure of the GAIN, there are hundreds of candidate tailoring variables at baseline that can be used to make the stage 1 services decision. At stage 2, the same baseline variables—as well as change in these measures from baseline to the end of month 3 and treatment assigned during the first 3 months—form a large list of possible tailoring variables from which to choose. Consequently, AIs generated from the GAIN have the possibility of being more individually-tailored than the template example provided in Fig. 1.

#### Outcomes

Outcomes of interest will be drawn from and informed by the set of “positive” measures selected by SAMHSA for the NOMs: i.e., no substance use, no SUD symptoms, no physical health problems, no mental health problems, no illegal activity, no justice system involvement, housed in the community, no family problems, vocationally engaged, and socially supported [76]. Each NOM will be turned into a “positive” binary indicator, and the total count of the number of positively endorsed NOMs will be used as our primary outcome variable. In addition to using the overall count of binary indicators of NOMs, we will also carefully examine continuous versions of these outcomes.

#### Analytic plan

A number of state-of-the-art methods will be utilized to identify candidate AIs using the CSAT dataset as part of our Aim 2 efforts. All of the methods we propose to use are based on the principles of Q-learning (Q-L)—an idea borrowed from computer science that can be seen as an extension of moderated regression analysis to the sequential decision-making setting [18, 61]. Q-L uses a backward induction logic (dynamic programming [77]) that incorporates effects of future decisions in evaluating present decisions. This ensures that the constructed AIs optimize outcomes over the short- and long-term, rather than selecting the setting at each stage that improves outcomes only in the short-term and, therefore, ignores any potentially beneficial delayed effects of earlier decisions. In the Q-L regression analyses, the goal will be to find AIs that maximize the mean number of positively endorsed NOMs (primary outcome) and continuous versions of individual NOMs (secondary outcomes). We will carefully compare and contrast the findings from various methods for implementing Q-L with our dataset. Please see Additional file 1 for a technical overview of Q-L.

#### Data partitioning and power

We will partition the CSAT dataset (N = 15,656) into two datasets, each of which is representative of all participants in the CSAT dataset. One dataset will be used for estimating AIs (Aim 2; e.g., our way to generate hypotheses about specific candidate AIs that might prove promising for adolescents) and a second dataset will be used for evaluation (see Aim 3 below). The purpose of data partitioning is to avoid “overfitting” by evaluating the identified AI with the same data used to empirically identify candidate AIs (Aim 2), a practice that may overstate the usefulness of an AI. Since Aims 2 and 3 will use mutually exclusive, random, subsamples of the complete CSAT dataset, power for both analyses is based on a raw sample size of approximately 7828 youth. Although a sample of 7828 youth is large, given the complexity of the AI estimation methods, it is difficult to judge the power of the analysis to identify meaningful tailoring variables or accurately distinguish among alternative treatment settings. Hence, the value of identified AIs will be assessed through out-of-sample evaluation in Aim 3. For Aim 3, assuming (conservatively) that a minimum of 500 youth in the data provide information about the effectiveness of a particular AI, and that weighting (discussed below) reduces this sample to an effective sample size of 250, we will be able to detect differences that are at least as large as 13 percentage points for binary outcomes and 0.25 effect sizes for continuous outcomes, assuming a two-tailed hypothesis test and a type-I error of 0.05. Mean rates of missingness on items ranged from less than 1 to 27.1%, with a mean of 13.4% across items at all of the follow-ups. Missingness will be dealt with explicitly by chained equations multiple imputation, [78], as implemented in the R package mice [79].

### Aim 3. Evaluate the relative effectiveness of candidate AIs

#### Objective

The third aim of this project is to evaluate the relative effectiveness of candidate AIs generated in Aim 2 by independently examining their causal effects on relevant clinical outcomes. In addition to comparing the relative effectiveness of candidate AIs with each other, we will also compare them against the following non-adaptive decision rules that are commonly used in everyday practice: (1) outpatient services for months 0–3 and then no treatment for months 0–6, (2) always outpatient services (i.e., outpatient for all months 0–6), (3) always intensive outpatient, (4) always residential\inpatient, and (5) no treatment for all months. As with Aim 2 analyses, the goal will be to find AIs most effective on the mean number of positively endorsed NOMs (primary outcome) and continuous versions of individual NOMs (secondary outcomes).

#### Analytic plan

To compare the relative effectiveness of candidate AIs against each other and non-adaptive decision rules, we will use marginal structural modeling (MSM): a class of causal longitudinal models for conceptualizing and estimating the causal effects of time-varying treatments [43, 80–84]. MSMs, when used together with “inverse-probability of treatment weighting” (IPT) [80, 83–86], can remove or greatly eliminate the bias that time-varying confounders contribute to estimates of the causal effects of the AIs. Estimated IPT weights for each of the adolescents in the dataset at each decision stage reduce the compositional imbalance in the confounders among adolescents who receive different sequences of treatments. We aim to generalize this analytic approach to allow for comparison between multiple AIs [87].

## Discussion

This project aims to provide several innovative insights for the field of adolescent substance use treatment. First, despite the obvious need and early successful efforts toward treatment guidelines, little to no research is currently and explicitly devoted to the empirical construction of AIs for guiding the individualized selection of settings for adolescent substance use treatment. The field has recognized the need to move beyond research that informs how to match adolescents with SUDs to initial treatments (i.e., based solely on known baseline characteristics of the adolescent at treatment entry) to research that informs how to adapt and re-adapt subsequent treatment over time to the specific needs of the individual [15, 19, 25]. The overarching scientific goal of this project is to begin the development of such guidance via the explicit development of empirically-based AIs. Second, stakeholder input is a critical supplement to the development of AIs that are both effective and feasible in practice. Existing conceptual models or behavioral theories are by themselves informative yet insufficient for constructing the data analysis models needed to identify effective AIs. To our knowledge, this project will be the first to use a mixed-methods, iterative approach whereby input from providers, policy-makers, researchers, and parents will directly inform the statistical models used to learn about effective and realistic AIs. Lastly, we will extend and illustrate the use of modern statistical methods for constructing and evaluating AIs using practice-based observational data. Methodologists have developed rigorous and effective methods such as Q-L, decision lists, and MSM for constructing and evaluating AIs from observational study data [43, 45, 88]. Despite the power, elegance, and availability of such methods, they are not routinely employed in adolescent substance use research.

We note several important considerations at the outset of this project. First, the robustness of findings from Aim 1 will depend on the stakeholders we are able to recruit for the panel and retain through the final rating round. Second, Aims 2 and 3 rely on data collected in the CSAT dataset, which provides rather coarse information about the timing of treatments received during a particular 90-day window. Because it is impossible to disentangle the order of treatment and outcomes within a given 90-day period in our dataset, finer-grained decision stages cannot be considered. Although it would have been ideal to have more detailed data on the timing of treatment receipt versus outcomes, we will appropriately lag our data to address this issue. In addition, we are limited in this study to the creation of AIs for assignment to different settings of treatment and cannot examine specific clinical interventions (e.g., cognitive behavioral therapy, motivational enhancement therapy) received in these settings, based on the availability of data in the CSAT dataset. Lastly, given that this project uses observational rather than experimental data, results generated are better viewed as hypothesis generating than confirmatory analyses. As such, this project represents an important first step in a process that itself will be adapted overtime.

In conclusion, this project will fill a major gap in adolescent addiction science and clinical practice. It expands upon the use of multi-dimensional assessments for substance use treatment planning by creating sequential, individualized decision rules for the setting of adolescent substance use treatment—supported by both stakeholder input and statistical evidence. Results from this project could be used to inform the further development and revision of proposed multi-dimensional assessment and treatment planning tools in the field of substance use—such as CONTINNUM, The ASAM Criteria Decision Engine™, and the GAIN Recommendation and Referral Summary). More immediately, identifying candidate AIs that have the promise of more effectively guiding decisions to move adolescents between outpatient, intensive outpatient, residential, and inpatient settings will provide guidance to current practitioners and lay the foundation for subsequent experiments that can test candidate AIs in rigorous clinical trials.

## Additional file

**Additional file 1.** Technical aspects of Aims 2 and 3.

## Abbreviations

AI: adaptive intervention
ASAM: American Society of Addiction Medicine
CSAT: Center for Substance Abuse Treatment
IPRAS: inter-percentile range adjusted for symmetry
IPT: inverse-probability of treatment weighting
MSM: marginal structural modeling
NOM: National Outcome Measure
Q-L: Q-learning
SAMHSA: Substance Abuse and Mental Health Services Administration
GAIN: Global Appraisal of Individual Needs
SUD: substance use disorder
