# Supplementary material for: Developing adaptive interventions for adolescent substance use treatment settings: protocol of an observational, mixed-methods project
Source: Addict Sci Clin Pract. 2017 Dec 19;12:35. doi: 10.1186/s13722-017-0099-4 (PMC5735877; doi:10.1186/s13722-017-0099-4)
Supplement: Supplementary file 1 — Additional file 1. Technical aspects of Aims 2 and 3. [file 13722_2017_99_MOESM1_ESM.docx]

**Additional file 1: Technical aspects of Aims 2 and 3**

**Aim 2 Analytic Plan.**

***Overview of Aim 2 Analytic Plan****.* A number of methods will be utilized to identify candidate AIs using the GAIN dataset as part of our Aim 2 efforts. All of the methods we propose to use are based on the principles of Q-learning. Q-learning, an idea borrowed from computer science, can be seen as an extension of regression analysis to the sequential decision-making setting.

To illustrate the key ideas behind QL, here we describe it in the context of linear regression since this version of QL can be thought of as a basis for all of the other methods we consider. Our description below closely follows QL as described in [1, 2]. Following this, we briefly describe how the other methods are variations of this approach.

For simplicity, first consider making the decision between outpatient and residential services based on maximizing the subsequent count of the number of positively endorsed NOMs outcomes a youth achieves. Suppose that for adolescents with a low NOMs count, residential services improves the long-term total count of positive NOMS outcomes (e.g., reduced substance use, reduced emotional problems, improved stability at home) more than outpatient services; whereas for adolescents with a high NOMs count, outpatient and residential services are about equally effective. In this simple case, the recommended decision is to provide residential services for youth with low NOMs counts, but to recommend the set of outpatient or residential services for youth with higher NOMs counts. The methods we will be considering quantify and extend this idea to multiple decision stages, for multiple candidate tailoring variables, and as a function of both short- and long-term outcomes.

QL uses a backward induction logic (dynamic programming [3]) that incorporates effects of future services decisions in evaluating present services decisions. This ensures that the constructed AIs optimize outcomes over the short- and long-term, or over the long-term, rather than selecting the treatment service option at each stage that improves outcomes only in the short-term and, therefore, ignores any potentially beneficial delayed effects of earlier decisions. In terms of notation, the complete data on each adolescent is $(O_{0},A_{1},O_{1},A_{2},O_{2},O_{3})$. $O_{0}$represents the baseline observations. $A_{j}$ represents the observed stage $j$ intervention services decision (outpatient, intensive outpatient, residential\inpatient, or no treatment); and $O_{j}$ ($j>0$) represents other observations collected during the $j$th interval that could either be evaluated for their use as tailoring variables in the AI (e.g, measures in $(O_{0},O_{1})$ could be evaluated as candidate tailoring variables for the services decision $A_{2}$) or as outcomes (e.g., $O_{3})$. There are 4 intervals in our data corresponding to: data collected at baseline measuring information about the adolescent prior to entering services (*j=0*), and data collected during months 0-3 (*j = 1*), 3-6 (*j = 2*), and 9-12 (*j = 3*) about services received and other outcomes during the given 3 month interval. Note that $(A_{1},O_{1})$ are collected concurrently during the *j=1* interval; and, similarly, ${(A}_{2},O_{2})$ are collected concurrently during the *j=2* interval. Also note that any observations past baseline ($O_{j} , j>0$) can be impacted by prior treatment services.

***The longitudinal outcome*.** The longitudinal outcome *Y_j_* quantifies the goal of the AI. In the Q-learning regression analysis, the goal will be to find an AI that maximizes the mean of this outcome (the outcome will be coded as higher is better). In the notation above, Y_j_ is part of O_j_, j = 2, 3. For illustration, let Y_j_ be the sum of (the time j) NOMS measures that the adolescent achieved over the course of the 3-month follow-up (eg., reduced substance use, reduced emotional problems, improved stability at home).

As discussed in the main narrative of the manuscript, other analyses will take use different forms of Y_j_. Each conceptualization of the longitudinal outcome could lead to a different AI.

***Q-Learning regression.*** We give a brief illustration of Q-learning (Q-L) using the above Yj as the outcome. A regression analysis is conducted for each stage, working backwards. In the stage 2 regression (the first regression done), the dependent variable is $Y_{3}$ (NOMS outcomes achieved between the 9 and 12-month follow-up visits). Independent variables include the stage 2 treatment indicator $A_{2}$ as well as individual variables collected prior to stage 2, denoted by $X_{2}$. Note that for simplicity, $A_{2}$ is assumed to be binary in this illustration (e.g., outpatient vs residential services); as stated above and in our manuscript, in our Aim 2 analyses, $A_{2}$ is multi-category. $X_{2}$ is a subset of the variables in $(O_{0},O_{1},A_{1},O_{2})$. $X_{2}$will include the variables suggested by stakeholders that are potentially useful in making the stage 2 treatment decision, as well as measures that are potentially highly correlated with $Y_{3}$. Thus, the regression model at stage 2 could be: $Y_{3}{=\beta}_{20}+\beta_{21}X_{2}+\beta_{22}A_{2}+\beta_{23}X_{2}A_{2}+\epsilon_{2}=Q_{2}+\epsilon_{2}$. The Q-Function, $Q_{j}$, is the part of the regression model at stage *j* that does not include the error term; the “Q” denotes average Quality [1]. A good treatment service at a particular stage is the treatment that produces the highest value of the Q-Function for that stage. Here, in the stage 2 regression, the Q-Function $Q_{2}$provides an assessment of the quality of a treatment service $A_{2}$ (on average) in terms of the outcome $Y_{3}$.

Next is the stage 1 regression which is similar to the stage 2 regression except for how the dependent variable is defined. The dependent variable here is an adjustment to $Y_{3}$, denoted $\tilde{Y}_{3}$, which is used to account for having made the best treatment decision at stage 2 plus *Y_2_*, which is the outcome during the 3-6 month interval (i.e., drawn from *O_2_*). We denote the dependent variable as $\tilde{Y}_{2}={Y_{2}+\tilde{Y}}_{3}$. The independent variables in the stage 1 analysis include the stage 1 treatment indicator $A_{1}$ and any pre-treatment variables $X_{1}$. $X_{1}$ includes variables in $O_{0}$ that are thought to be potentially useful in making the stage 1 treatment decision, as well as measures that are highly correlated with $\tilde{Y}_{2}$. The stage 1 regression may look like this: $\tilde{Y}_{2}=\beta_{10}+\beta_{11}X_{1}+\beta_{12}A_{1}+\beta_{13}X_{1}A_{1}. +\epsilon_{1}$ = $Q_{1} +\epsilon_{1}$. Here, in this stage 1 regression, the Q-Function $Q_{1}$provides an assessment of the quality of a treatment service $A_{1}$ (on average) taking into account both the short-term outcome ($Y_{2}$) and long-term outcome we expect to achieve by making more optimal decisions in the future ($\tilde{Y}_{3}$). Note that if $\tilde{Y}_{2}=\tilde{Y}_{3}$, then $Q_{1}$ would provide an assessment of the quality of $A_{1}$ taking into account only the long-term outcome we expect to achieve by making more optimal decisions in the future ($\tilde{Y}_{3}$).

From a technical point of view, the stage 2 Q-Function is $Q_{2}\left( x_{2},a_{2} \right)=E(Y_{3}|X_{2},A_{2})$ and the Q-Function at stage 1 is $Q_{1}\left( x_{1},a_{1} \right)=E(Y_{2}+\max_{a_{2}} Q_{2}\left( x_{2},a_{2} \right)|X_{1}=x_{1},A_{1}=a_{1})$ and the “best” intervention service at stage $j$for an adolescent with data $x_{j}$ is given by $d_{j}(x_{j})=\underset{a_{j}}{arg max} Q_{j}\left( x_{j},a_{j} \right)$. The Q-Learning constructed AI is given by the sequence of decision rules ${d=(d}_{1}(x_{1}),d_{2}(x_{2}))$.

The version of QL we propose to use is an extension of the above-described method to multiple treatment at each stage; that is, where *A_j_* is multicategory. In addition, note that the estimated best AI is also dependent on the choice of model Q_j_ at each stage, which in the examples above, were taken to be linear in the unknown parameters $\beta_{2}$. For this (and other) reasons, various approaches have been proposed that extend the above to allow for more flexibility in modeling. We propose to examine some of these extensions; namely, those for which there exists easy to implement software and which allow for multicategory treatments at each stage. These include penalized Q-learning [4], and DecisionLists [5, 6]. These two methods generalize Q-learning in terms of the type of regression models they employ. Instead of fitting a standard linear regression model like $\tilde{Y}_{2}=\beta_{10}+\beta_{11}X_{1}+\beta_{12}A_{1}+\beta_{13}X_{1}A_{1}+\epsilon_{1}$, these other methods allow for a penalized regression model (penalized Q-L) or a nonparametric model (DecisionLists). Because of the large number of potential tailoring variables at multiple stages and interacting with multiple treatments, selecting only important variables (as done in penalized Q-L) to include in the regression model yields more interpretable AIs and may lead to improved performance and lower variability than standard Q-L. Similarly, DecisionLists are particularly attractive given that they provide much more flexible and interpretable AIs while also selecting important variables.

We will separately apply each method to the (training) GAIN data to generate candidate AIs. We will then use the (evaluation) GAIN data to carefully compare and contrast the findings from each method and quantitatively assess the relative performance of the identified candidate AIs.

**Aim 3 Analytic Plan.**

***Overview of Aim 3 Analytic Plan*.** Marginal structural modeling (MSM) [7-12], a class of causal longitudinal models for conceptualizing and estimating the causal effects of time-varying treatments, will be used to compare the AIs. Relative to methods that do not account appropriately for time-varying confounders, MSMs, when used together with “inverse-probability of treatment weighting” (IPT) [8, 11-14] weights, can be used to obtain robust estimates of the causal effects of the AIs by removing or greatly eliminating the bias introduced from time-varying confounders. Taking again the example of the count of important NOMS measures as the outcome, we can treat this count as continuous for the purposes of demonstrating the MSM. Consider the following linear MSM for the population of adolescents represented in the CSAT data: $E \left( Y_{3}\left( d^{*} \right) \right)=\gamma_{0}+\gamma_{1}I(follow AI d^{*})$, where $Y_{3}$ represents the count of important NOMS during months 9-12, $d^{*}$ represents any one of the AIs constructed in Aim 2, and $I(follow AI d^{*})$ is a binary indicator for whether/not an adolescent’s data is consistent with AI $d^{*}$. In this example model, $\gamma_{0}+\gamma_{1}$ is the expected important NOMS count during months 9-12 had all adolescents in our population followed the AI given by $d^{*}$, and $\gamma_{1}$ represents the causal additive increase in the count of following the AI given by $d^{*}$versus not following it. In the above illustration, we used $Y_{3}$ as the outcome; however we will apply the above method with the longitudinal outcome $(Y_{2},Y_{3})$. We will also include key baseline covariates *O_0_* in the above MSM.

***Weighting to reduce time-varying confounding bias.*** IPT weights are necessary to estimate such MSMs to account for the potential for confounding bias due to time-varying variables that both affect why an individual received a treatment service and are affected by treatment. We will adjust for such time-varying confounding in this study via IPT weights. Conceptually, IPT weights reduce the compositional imbalance between treatment service groups due to the confounders. Let $C_{j}$ denote potential time-varying confounders derived from (O_0_, …, O_j-1_); that is, $C_{j}$ are variables assumed to affect both the choice of the observed treatment $A_{j}$ at stage $j$ as well as the proximal outcome $Y_{j}$ . For simplicity, suppose that there are only two intervention service options at each stage: $A_{j}=1$ or $A_{j}=0$. The IPT weights at stage $j$ are defined as $W_{j}\left( A_{j},C_{j} \right)={A_{j}}/{p(C_{j})}+{(1-A_{j})}/{(1-p(C_{j}))}$ where $p\left( C_{j} \right)=Pr\left( A_{j}=1 | C_{j} \right).$ An estimated weight is created for each of the adolescents at both of the stages of treatment in the data set. Then, each adolescent is assigned a final weight, which is the product of the weights across all stages of treatment: $W=\prod_{j=1}^{2} W_{j}$.

We will estimate MSMs similar to the above, but we will generalize the approach to allow for comparison between multiple AIs [15, 16]. We will improve statistical power in these comparisons by conditioning on baseline covariates taken from $O_{0}$ and employ the more efficient estimators [15-18]. Robust standard errors, which take into account the sampling variance in the estimation of the weights, will be used for making statistical inferences.

**References**

1. Nahum-Shani, I., et al., *Q-learning: a data analysis method for constructing adaptive interventions.* Psychol Methods, 2012. **17**(4): p. 478-94.

2. Nahum-Shani, I., et al., *A SMART data analysis method for constructing adaptive treatment strategies for substance use disorders.* Addiction, 2017. **112**(5): p. 901-909.

3. Bellman, R.E., *Dynamic Programming*. Dover Books on Computer Science. 2003, Mineola (NY): Dover Publications.

4. Qian, M. and S.A. Murphy, *Performance guarantees for individualized treatment rules.* Annals of Statistics, 2011. **39**(2): p. 1180.

5. Zhang, Y., et al., *Using decision lists to construct interpretable and parsimonious treatment regimes.* Biometrics, 2015. **71**(4): p. 895-904.

6. Zhang, Y., et al., *Interpretable Dynamic Treatment Regimes.* arXiv, 2016.

7. Hernan, M.A., B. Brumback, and J.M. Robins, *Marginal structural models to estimate the causal effect of zidovudine on the survival of HIV-positive men.* Epidemiology, 2000. **11**(5): p. 561-70.

8. Hernan, M.A., B.A. Brumback, and J.M. Robins, *Estimating the causal effect of zidovudine on CD4 count with a marginal structural model for repeated measures.* Stat Med, 2002. **21**(12): p. 1689-709.

9. Robins, J.M., *A new approach to causal inference in mortality studies with a sustained exposure period: Application to the healthy worker survivor effect.* Mathematical Modelling, 1986. **7**: p. 1393-1512.

10. Robins, J.M., *Addendum to “a new approach to causal inference in mortality studies with sustained exposure periods-application to control of the healthy worker survivor effect.* Comput Math Appl, 1987. **14**(9-12): p. 923-945.

11. Robins, J.M., *Association, causation, and marginal structural models.* Synthese, 1999. **121**: p. 151-179.

12. Robins, J.M., M.A. Hernan, and B. Brumback, *Marginal structural models and causal inference in epidemiology.* Epidemiology, 2000. **11**(5): p. 550-60.

13. Hernan, M.A., B. Brumback, and J.M. Robins, *Marginal structural models to estimate the joint causal effect of nonrandomized treatments.* J Am Stat Assoc, 2001. **96**(454): p. 440-448.

14. Robins, J.M., *Marginal structural models versus structural nested models as tools for causal inference*, in *Statistical models in epidemiology: The environment and clinical trials*, M.E.H.D. Berry, Editor. 1999, Springer: New York. p. 95-134.

15. Orellana, L., A. Rotnitzky, and J.M. Robins, *Dynamic regime marginal structural mean models for estimation of optimal dynamic treatment regimes, Part I: main content.* Int J Biostat, 2010. **6**(2): p. Article 8.

16. Orellana, L., A. Rotnitzky, and J.M. Robins, *Dynamic regime marginal structural mean models for estimation of optimal dynamic treatment regimes, Part II: proofs of results.* Int J Biostat, 2010. **6**(2): p. Article 9.

17. Murphy, S.A., et al., *Marginal Mean Models for Dynamic Regimes.* J Am Stat Assoc, 2001. **96**(456): p. 1410-1423.

18. Zhang, B., et al., *Web-based Supplementary Materials for "A Robust Method for EstimatingOptimal Treatment Regimes"*.
